# Road Traffic Noise Exposure and Birth Outcomes: An Updated Systematic Review and Meta-Analysis

**DOI:** 10.3390/ijerph16142522

**Published:** 2019-07-15

**Authors:** Angel M. Dzhambov, Peter Lercher

**Affiliations:** 1 Department of Hygiene and Ecomedicine, Faculty of Public Health, Medical University of Plovdiv, 4002 Plovdiv, Bulgaria; 2 Institute for Highway Engineering and Transport Planning, Graz University of Technology, 8010 Graz, Austria

**Keywords:** birth weight, environmental noise, pregnancy outcomes, preterm birth, small for gestational age, transportation noise

## Abstract

Unlike the other WHO evidence reviews, the systematic review on birth outcomes could not provide a quantitative estimate of the effect of environmental noise. With that in mind, we aimed to update it with additional studies published through to 12 May, 2019 to allow for a formal meta-analysis of the association of residential road traffic noise with birth weight, low birth weight (LBW), small for gestational age (SGA), and preterm birth (PTB). The quality effects and random effects estimators were used for meta-analysis and the robustness of findings was tested in several sensitivity analyses. Nine studies were included in the qualitative synthesis, from which we extracted seven estimates for birth weight (*n* = 718,136 births) and LBW (*n* = 620,221), and five for SGA (*n* = 547,256) and PTB (*n* = 74,609). We found −8.26 g (95% CI: −20.61 g, 4.10 g) (*I^2^* = 87%) lower birth weight associated with a 10 dB(A) increase in day-evening-night noise level (L_den_), and this effect became significant in sensitivity analyses. No evidence of significant effects was found for LBW (OR = 1.06; 95% CI: 0.91, 1.23) (*I^2^* = 49%), SGA (OR = 1.02; 95% CI: 0.86, 1.21) (*I^2^* = 90%), or PTB (OR = 1.00; 95% CI: 0.79, 1.27) (*I^2^* = 69%). The quality of evidence for continuous birth weight was graded as “moderate”, while for the other outcomes it was deemed “very low”. Finally, we discuss limitations of the risk of bias assessment criteria employed by Nieuwenhuijsen et al.

## 1. Introduction

Road traffic noise, the dominant type of environmental noise in urban settings, has been linked to a multitude of adverse health outcomes, including annoyance [1], poor mental health [2], sleep disturbance [3], and cardiometabolic disorders [4]. Recently, this growing literature was systematically reviewed in a series of WHO evidence reviews, published in the International Journal of Environmental Research and Public Health, with the intent to inform policy and aid development of new health effects-based environmental noise guidelines for the WHO European region [5]. These reviews produced quantitative exposure-response functions for the majority of target outcomes [6]; however, the systematic review on birth outcomes [7] was constrained by the small number of compatible studies, and therefore, no meta-analysis was conducted. Nieuwenhuijsen et al. [7] reported “low quality evidence for an association between road traffic noise and low birth weight, preterm birth and small for gestational age”, but these results relied only on qualitative literature synthesis. As the authors correctly noted [7], one earlier meta-analysis [8] focused on occupational noise studies, but did not really tell us anything about road traffic noise. Quantitative evidence would be necessary for burden of disease estimation and should allow a comparison of road traffic noise with other competing risk factors for adverse birth outcomes.

Research on the subject is gaining momentum, and several new research results [9,10,11,12,13] were published after the last update of the WHO review in December 2016. With that in mind, we aimed to augment the WHO review [7] with additional studies to allow for a formal meta-analysis of the association of residential road traffic noise with birth outcomes. To ensure methodological continuity and comparability, we largely adhered to the systematic review protocol of the WHO evidence paper [7], with some modifications. We then went on to address some limitations of Nieuwenhuijsen et al.’s method.

## 2. Materials and Methods

### 2.1. Systematic Review Protocol

The literature searches were carried out independently by both authors, following the Preferred Reporting Items for Systematic Reviews and Meta-Analyses (PRISMA) [14] guidelines. Only minor disagreements needed to be resolved by discussion.

We considered all relevant studies already included in the WHO review [7]. That list was augmented with studies that we were aware of based on our expert knowledge of respective literature on traffic noise and birth outcomes. Further, the identification of studies published after the WHO review [7] was refined by a systematic literature search for original research published in English in the period 2016–2019 (last updated on 12 May, 2019). We searched MEDLINE (PubMed) and EMBASE (ScienceDirect) using the keyword string: ((“road traffic noise” OR “traffic noise”) AND (“birth” OR “pregnancy” OR “preterm” OR “small for gestational age”)). No additional filters were applied. Finally, we contacted the authors of several potentially relevant studies (e.g., [15]) who could provide useful effect estimates by re-analyzing their data.

The inclusion criteria we adopted were largely consistent with those developed for the WHO review [7]. However, we wanted to narrow down the scope of our review to road traffic noise and commonly reported birth outcomes (birth weight, low birth weight; LBW, small for gestational age; SGA, and preterm birth; PTB), for which a sufficient number of comparable effect estimates was likely to exist. Moreover, unlike the WHO review [7], we considered only cohort, case-control, cross-sectional, and ecological study designs that could be pooled together in a meaningful way; thus, time-series studies (e.g., [16,17]) were excluded.

### 2.2. Data Extraction

Information was extracted from each retrieved article on: (1) Number of distinct datasets analyzed; (2) study design; (3) sample size and maternal characteristics; (4) outcome definition and assessment; (5) exposure definition and assessment; (6) statistical analysis; (7) adjustments; (8) and crude and adjusted effect size estimates. To enable comparison with previous meta-analyses on health effects of road traffic noise [6], we extracted effect estimates (unstandardized linear regression beta coefficients or odds ratios) rescaled to a 10 dB(A) increment in noise exposure. If estimates were scaled to another unit increase in road traffic noise (e.g., 6 dB in [18]; 3.5 dB in [10]; 6.7 dB in [19]; 5 dB in [9]), they were transformed as needed using the expressions “exp((ln(reported effect estimate)/original unit increase)*10)” for odds ratios and “(reported effect estimate/original unit increase)*10” for beta coefficients.

Most studies used the noise indicator day-evening-night noise level (L_den_), therefore, we adopted it for reporting our findings. Of note, since results were expressed as linear exposure-response relations, no conversion of other indicators to L_den_ was necessary because the slopes would not be affected by this linear conversion [20,21].

Some data extraction decisions required justification. For example, Dzhambov et al.’s [13] article reported results for two cross-sectional surveys that systematically differed on area-level factors. Therefore, we treated them as separate studies in our meta-analysis. In contrast, Markevych et al. [15] pooled together two compatible datasets. After discussing the appropriate treatment of the data with Dr. Markevych, it was decided to use the combined effect estimate in the meta-analysis. Second, careful inspection of the results reported by Nieuwenhuijsen et al. [12] revealed an unrealistic estimate of the effect of continuous noise exposure on low birth weight. The authors confirmed that, indeed, a typo was involved and provided the correct estimate. Third, for one of the sensitivity analyses (estimates adjusted for NO_2_) we used GetData Graph Digitizer v. 2. 26 (© 2002–2013, S. Fedorov) to extract effect estimates and their 95% CIs from a figure reported in the article by Gehring et al. [18]. Fourth, because only a few studies (e.g., [18,22]) reported both trimester-specific and entire pregnancy noise exposure, we considered the latter to ensure comparability with other studies. For the same reason, when a study reported results for both 24-h and night-time noise level (e.g., [12]), we considered the former. Finally, although some studies (e.g., [9]) investigated combined noise exposure, they were retained because the authors asserted that road traffic was the dominant noise source in the study area (cf. [23]).

### 2.3. Risk of Bias Assessment

To ascertain the validity of eligible studies included in the qualitative synthesis, a list of predefined safeguards was used to avoid bias related to different study characteristics. This scale was previously developed for the WHO review [7]. In one of the sensitivity analyses, we provide an alternative view on study bias by using modified criteria and scoring (Appendix A, Table A1).

### 2.4. Meta-Analysis

The unstandardized linear regression coefficient and the odds ratio were selected as measures of effect. We pooled exposure-response relationships between a 10 dB(A) increase in L_den_ and (1) continuous birth weight, (2) LBW, (3) SGA, and (4) PTB. Effect estimates were pooled under two alternative meta-analytical estimators—the random effects (RE) model and the quality effects (QE) model. While meta-analysts typically use the RE estimator when facing high between-study heterogeneity, it has been scrutinized on multiple occasions [24,25,26,27,28] for underestimating the true variance and producing overconfident results. Therefore, we report our main findings under the QE model, which allows the inclusion of information on the risk of bias in primary studies into the estimation of meta-analysis weights [27]. It favors larger studies with lowest probability of random error and exhibits a lesser true variance than the RE model, regardless of heterogeneity, thereby maintaining the correct coverage probability of the confidence interval without underestimation of the statistical error. Following Nieuwenhuijsen et al. [7], we based our main models on effect estimates adjusted for relevant confounders, but not for air pollution (wherever possible).

We assessed the possibility of publication bias by evaluating Doi plots [29]. The Doi plot replaces the conventional scatter (funnel) plot of precision versus effect with a variant of the normal quantile versus effect plot by using a rank-based measure of precision (Z score), instead of the standard error, and plots it against the effect size [29,30]. The most precise studies define the midpoint around which results scatter, whereas smaller less precise studies produce an effect size that scatters increasingly widely, and the absolute Z score gradually increases for both smaller and larger effect sizes on either side of that of the precise studies. If the studies are homogeneous and not affected by selection or other forms of bias, the plot resembles a symmetrical mountain with a similar number of studies and equal spread on each side. Otherwise, asymmetry exists [30]. Doi plot asymmetry was quantified with the Luis Furuya-Kanamori (LFK) index by averaging half of the sum of the Z score plus the normalized effect size across the meta-analysis [29,31]. The LFK index quantifies the difference between the two areas under the Doi plot, created by the perpendicular line to the X-axis from the effect size with the lowest absolute Z score on the Doi plot [29]. The accuracy of the LFK index in discriminating between asymmetry due to publication bias versus chance/no asymmetry is superior to that of Egger’s test, particularly when the number of studies is small (sensitivity of 72% vs. 19% in a meta-analysis of 5 studies) [29]. A symmetrical, mountain-like Doi plot and LFK index <|1| indicates no asymmetry, LFK index between |1| and |2|, minor asymmetry, and LFK index >|2|, major asymmetry [30].

Several sensitivity analyses complemented the main meta-analysis. First, we used the leave-one-out method to check the robustness of the pooled effect estimate upon one-at-a-time exclusion of each individual study estimate. Second, our alternative bias scores were fed to the QE algorithm and the main models were repeated. Finally, we determined the impact adjusted for covariates had on results by pooling separately crude and air pollution-adjusted effect estimates.

Statistical heterogeneity was indicated by a significant Cochran’s Q at the *p* <0.1 level, *I^2^* >30%, and/or tau-squared >1. Meta-analyses were conducted in MetaXL v. 5.3 (EpiGear International Pty Ltd., Sunrise Beach, Queensland, Australia).

### 2.5. Quality of Evidence Assessment

The quality of evidence for the effect of road traffic noise on each outcome was rated according to the GRADE system [32]. Mimicking the modified scoring method of the WHO evidence review on traffic noise and cardiometabolic outcomes [4], cohort and case-control started with a “high quality” rating because a randomized controlled trial is neither a typical nor feasible design in the field; on the other hand, analytic studies are considered the gold standard (in practical terms) [4]. The quality of evidence was reduced by one category by high risk of bias (high bias across studies), inconsistency of results (heterogeneity and disparate findings across studies), indirectness of evidence, imprecision of the effect estimate (wide 95% CI), publication bias, or when the evidence was based on only one high quality study. If not previously downgraded, the quality of evidence was increased by one grade if the magnitude of the effect was large, if accounting for all plausible biases would have increased the observed effect, or if there was an exposure-response gradient (significant trend) [21].

## 3. Results

### 3.1. Literature Search Results

**Figure 1:**
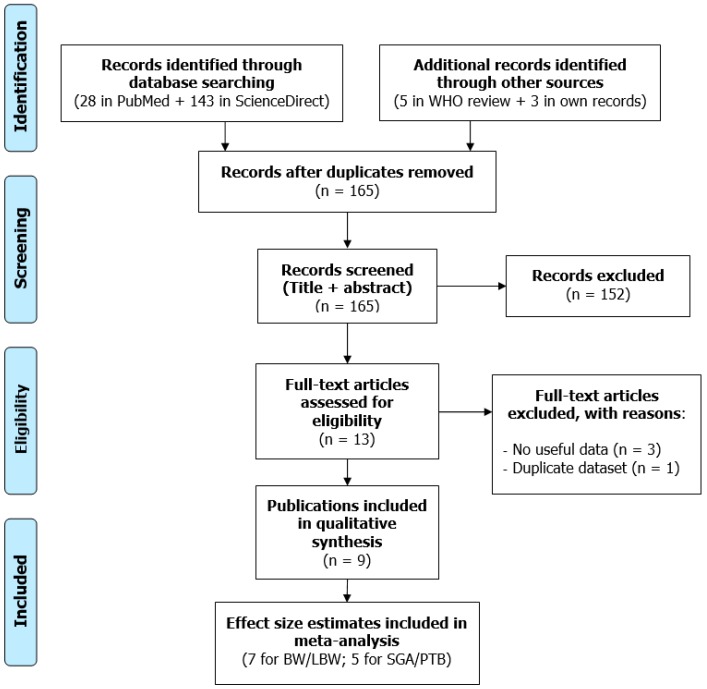
Study selection flow diagram (BW/LBW: Birth weight/Low birth weight, SGA/PTB: Small for gestational age/Preterm birth).

The study selection flow diagram is presented in Figure 1. Database searches identified 28 records in PubMed and 143 in ScienceDirect. The WHO review [7] provided five publications published before 2017 [18,19,22,33,34]. We had prior knowledge of the study of Barba-Vasseur et al. [9]. In addition, we asked authors of potentially relevant studies [15,35] to re-analyze their datasets. Markevych et al. [15] had examined the effect of greenness on birth weight, but they were able to estimate the effect of road traffic noise.

After removing duplicate records, we screened the titles and abstracts of the remaining 165 records and further excluded 152 that were deemed irrelevant, leaving us with 13 full-texts for in-depth review. Three of them were discarded because they contained no useful data—Wu et al. [34] did not report an effect estimate, and Cusack et al. [35] and Weyde et al. [36] did not respond to our request to re-analyze their datasets. We dropped one study because it was based on the same dataset as another already included study [33]. Thus, nine publications were finally included in the qualitative synthesis [9,10,11,12,13,15,18,19,22], with some of them supplying more than one effect estimate.

### 3.2. Narrative Description of the Studies Included

Table 1 shows abstracted descriptive characteristics of the studies included in the systematic review. Of the nine publications, six reported results from cohort studies [10,11,12,18,19,22], one from a case-control study [9], and three from cross-sectional studies [13,15]. Of note, Dzhambov et al. [13] analyzed two distinct datasets (the Unterinntal (UIT) and Brenner Base Tunnel (BBT) surveys), therefore, we treated them as independent studies. All studies but one [18] were conducted in Europe.

Overall, most authors considered singleton live births. The design of Dzhambov et al.’s [13] studies, however, did not allow ascertaining that twin pregnancies were completely excluded; still, the number of twin pregnancies was deemed to be reasonably low. Some studies included only term babies [10,12,15,19,22], whereas others [9,11,13,18] included both term and preterm babies. Importantly, all newborns included by Markevych et al. [15] had normal birth weight. Some studies considered multiple birth outcomes—there were seven studies on continuous birth weight [10,12,13,15,18,22]; seven on LBW [10,11,12,13,18,19]; five on SGA [10,13,18,19]; and five on PTB [9,11,13,18]. All outcomes were defined based on official medical records.

All studies calculated road traffic noise exposure from a European Union noise map or by another valid method. The majority of studies calculated noise level at the most exposed façade [10,11,15,22], but Gehring et al. [18] calculated noise at postal code-level, and Dadvand et al. [19]— at street-level. Input data were generally of acceptable quality, but only one study considered floor of the dwelling [9] and one had data on noise barriers [15]. Noise measurement validation was conducted in four studies [9,10,13]. L_den_ was the indicator of choice in most studies, the only exceptions being Smith et al. [10] who reported L_Aeq,16hr_ and the UIT survey where L_dn_ was used [13]. Mean/median noise level varied from 46 dB in the UIT survey [13] to 69 dB in the study of Dadvand et al. [19]. The noise range in the study of Gehring et al. [18] was exceptionally wide. It should be noted that only four studies [11,18,19,22] had information on maternal residential history during pregnancy and were able to calculate time-varying noise exposure; conversely, only the residential address at delivery was known in three studies [9,10,12]; and noise data were available at a later point in time after delivery in three studies [13,15]. In the latter studies [13,15], noise level did not exactly represent noise exposure during pregnancy, therefore, they were regarded as purely correlational.

Statistical methods were compatible across studies—the effect on birth weight was tested with linear regressions and expressed as unstandardized beta coefficients, and for dichotomous outcomes the authors used logistic regressions and reported odds ratios. Five of the studies [10,12,18,19,22] considered important maternal and pregnancy-related confounding factors, including age, education, socioeconomic position/income, smoking during pregnancy, parity, gestational age, pre-pregnancy BMI, and time of the year. On the other hand, Barba-Vasseur et al. [9] had information only on maternal age, parity and smoking, and three studies [11,13,15] did not account for some of the confounders. Importantly, although Smith et al. [10] considered several factors, they were measured only at area-level. In terms of results, Gehring et al. [18] reported significantly lower birth weight, higher odds of LBW, and SGA with increasing noise level; Smith et al. [10] found significant associations with birth weight and SGA; and Dzhambov et al. [13] with LBW in the UIT survey. Most of the other findings also went in the hypothesized direction, but failed to meet the formal level of statistical significance.

According to the bias scores presented in Table 2, the risk of bias was “low” in the majority of studies and “unknown” in three [13,15]. The latter were penalized for their correlational design and temporal mismatch between exposure and outcome measures. Least bias was suspected for the studies of Gehring et al. [18], Dadvand et al. [19], and Hjortebjerg et al. [22]. Alternative bias scoring is presented in Appendix A, Table A2.

### 3.3. Main Meta-Analysis

**Figure 2:**
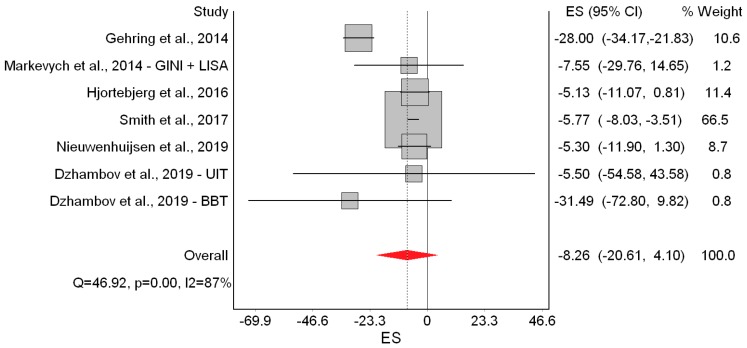
Forest plot showing the effect of a 10 dB(A) increase in road traffic noise level on continuous birth weight under the quality effects model (ES: Effect size, CI: Confidence interval, Q and I2 (I^2^): Heterogeneity statistics).

**Figure 3:**
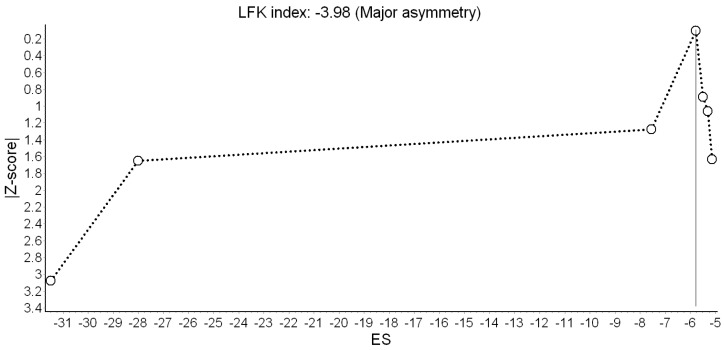
Doi plot showing the risk of publication bias in the meta-analysis of the association between road traffic noise and continuous birth weight (ES: Effect size, LFK: Luis Furuya-Kanamori index).

Results of the QE meta-analysis for birth weight are shown in Figure 2. Based on seven estimates, a 10 dB(A) increase in L_den_ was (marginally) associated with −8.26 g lower birth weight. Heterogeneity in the model was high, but completely due to the study of Gehring et al. [18]. Visual inspection of the Doi plot indicated major asymmetry (Figure 3). Coupled with the high LFK index (−3.98), that suggested that publication bias was likely. However, it was almost completely due to the BBT survey [13] whose exclusion reduced the asymmetry to minor (LFK = −1.14). Under the RE model, the study weights were distributed more evenly and the pooled effect became significant (*β* = −11.22 g; 95% CI: −19.75 g, −2.69 g).

Next, we repeated the QE meta-analysis by excluding each study one-at-a-time. This only changed the picture when the study of Gehring et al. [18] was excluded, which reduced the I^2^ to 0% and increased the precision of the point estimate yielding a significant pooled effect (*β* = −5.95 g; 95% CI: −8.03 g, −3.86 g). Finally, using our modified bias scores, the pooled effect was *β* = −9.23 g (95% CI: −20.11 g, 1.65 g). (Appendix A, Table A3)

**Figure 4:**
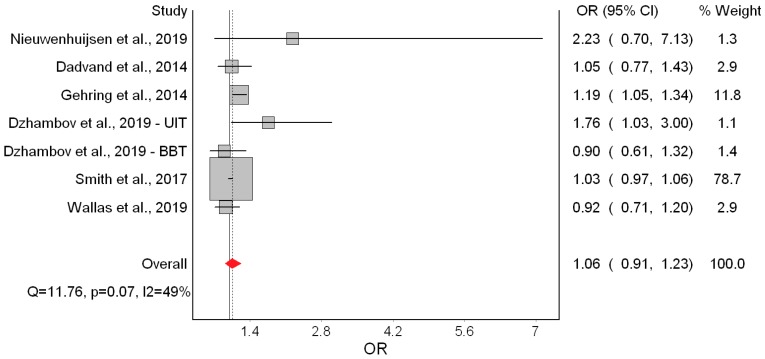
Forest plot showing the effect of a 10 dB(A) increase in road traffic noise level on low birth weight under the quality effects model (OR: Odds ratio, CI: Confidence interval, Q and I2 (I^2^): Heterogeneity statistics).

**Figure 5:**
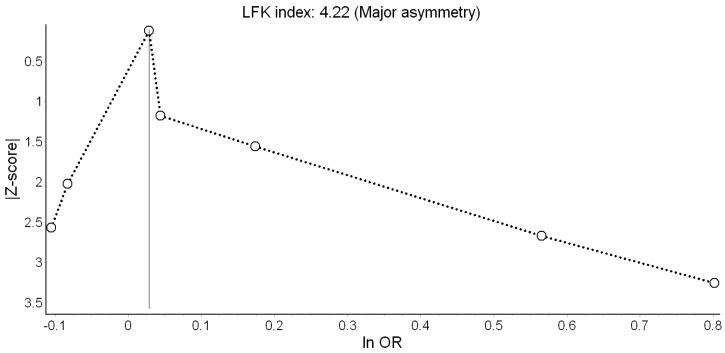
Doi plot showing the risk of publication bias in the meta-analysis of the association between road traffic noise and low birth weight (ln OR: Log odds ratio, LFK: Luis Furuya-Kanamori index).

Only two [18,19] of the seven studies indicated significantly higher odds of LBW (Figure 4). Overall, the QE model yielded non-significant odds of LBW of 1.06 associated with a 10 dB(A) increase in L_den_. This effect appeared driven by Smith et al. [10]. Heterogeneity in this model was moderate and mostly due to the study of Gehring et al. [18]. Major asymmetry in the Doi plot and LFK index of 4.22 suggested presence of publication bias (Figure 5). The RE estimator produced a materially identical result (OR = 1.08; 95% CI: 0.96, 1.21).

The only remarkable results of the leave-one-out meta-analysis were the low heterogeneity (*I^2^* = 25.94%) when Gehring et al. [18] was excluded, and the doubling of the odds (OR = 1.15; 95% CI: 0.94, 1.40) when Smith et al. [10] was excluded. That, however, did not improve the precision of the estimate. Using the alternative bias scores did not change the picture (OR = 1.08; 95% CI: 0.95, 1.24). (Appendix A, Table A3)

**Figure 6:**
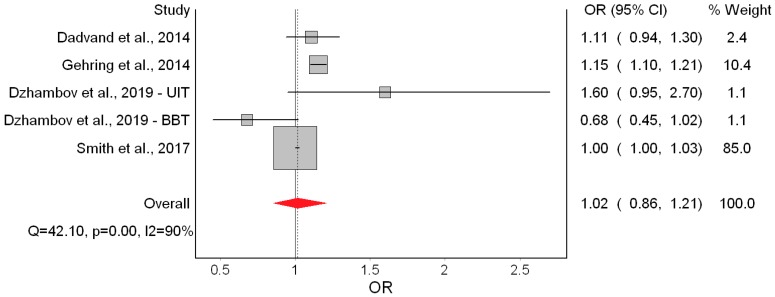
Forest plot showing the effect of a 10 dB(A) increase in road traffic noise level on small for gestational age under the quality effects model (OR: Odds ratio, CI: Confidence interval, Q and I2 (I^2^): Heterogeneity statistics).

**Figure 7:**
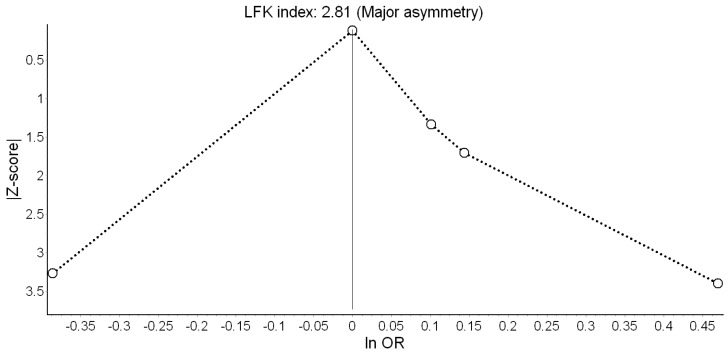
Doi plot showing the risk of publication bias in the meta-analysis of the association between road traffic noise and small for gestational age (ln OR: Log odds ratio, LFK: Luis Furuya-Kanamori index).

We observed no effect on SGA (Figure 6). There was high heterogeneity in the model and evidence of publication bias (Figure 7). The RE model led us to the same conclusion of no significant effect (OR = 1.07; 95% CI: 0.95, 1.20).

Smith et al. [10] was again the study with the largest weight. Its exclusion in the leave-one-out meta-analysis increased the pooled effect to OR = 1.15 (95% CI: 0.92, 1.43), but the confidence interval remained wide. Using alternative bias scores did not materially change the effect (OR = 1.03; 95% CI: 0.89, 1.20). (Appendix A, Table A3)

**Figure 8:**
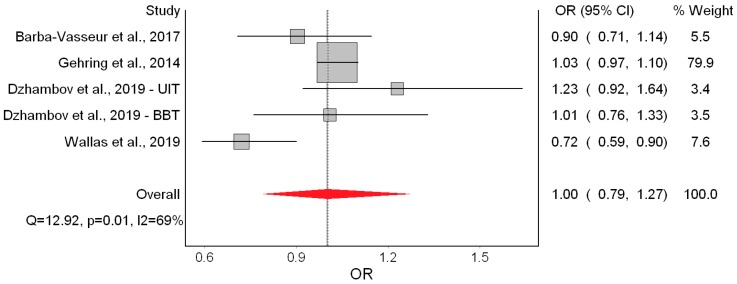
Forest plot showing the effect of a 10 dB(A) increase in road traffic noise level on preterm birth under the quality effects model (OR: Odds ratio, CI: Confidence interval, Q and I2 (I^2^): Heterogeneity statistics).

**Figure 9:**
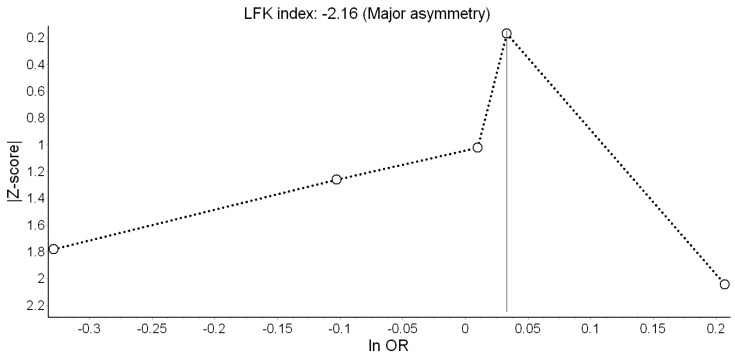
Doi plot showing the risk of publication bias in the meta-analysis of the association between road traffic noise and preterm birth (ln OR: Log odds ratio, LFK: Luis Furuya-Kanamori index).

Finally, the QE model did not indicate an effect on PTB (Figure 8). Heterogeneity in the model was high and due to the study of Wallas et al. [11]. The Doi plot revealed major asymmetry (Figure 9). This conclusion was robust to using the RE model (OR = 0.96; 95% CI: 0.82, 1.12), excluding individual estimates, and using alternative bias scores (OR = 1.01; 95% CI: 0.79, 1.27). (Appendix A, Table A3)

### 3.4. Crude and Air Pollution-Adjusted Effects

In the final sensitivity analysis, we pooled separately crude and air pollution-adjusted estimates to evaluate the impact of confounding factors in the model. Table 3 shows the results in these alternative scenarios. The associations between L_den_ and each of the birth outcomes showed a pattern of decreasing magnitude from crude to fully-adjusted models. For instance, the odds of LBW were significantly increased by 28% in the crude model, but this effect dropped to 1% (non-significant) in the air pollution-adjusted model. Overall, we observed only minor confounding by air pollution.

## 4. Discussion

### 4.1. Major Findings

We systematically reviewed the literature on residential road traffic noise and birth outcomes. Nine studies were included in the qualitative synthesis, from which we extracted seven estimates for continuous birth weight (*n* = 718,136 births) and LBW (*n* = 620,221 births), and five for SGA (*n* = 547,256 births) and PTB (*n* = 74,609 births).

In the main meta-analysis, we found −8.26 g lower birth weight associated with a 10 dB(A) increase in L_den_. No evidence of significant effects on the other birth outcomes was found, although non-significant trends in the expected direction were seen for LBW and SGA. Interestingly, the heterogeneity and publication bias in the birth weight model were completely due to Gehring et al. [18] and the BBT survey [13], respectively. One possible explanation for the contribution of Gehring et al. [18] to statistical heterogeneity could be that the L_den_ range in that study was exceptionally wide (6.2 to 89 dB(A)); such high contrast in participants’ exposure could account for the strong harmful effects seen in that study, compared with the others studies. As for the BBT survey, it was conducted in a unique setting (two Alpine valleys), across which heterogeneity in the effect of road traffic noise was attributed to varying contextual features of the area [13].

The quality of evidence for birth weight was graded as “moderate”; that is, the true effect of noise on birth weight was probably close to the estimated effect. The main rationale for this was the consistent effect observed across all studies in the expected direction. Furthermore, the risk of bias in four out of seven studies was “low” and heterogeneity and publication bias were completely due to Gehring et al. [18] and the BBT survey [13], both of which had modest contributions to the overall effect. Moreover, there was an exposure-response trend that became significant when the study of Gehring et al. [18] was excluded or when the RE model was employed. Regarding the other outcomes, we found indications of publication bias and/or heterogeneity, and inconsistent results across studies; therefore, the quality of evidence for LBW, SGA, and PTB was downgraded to “very low”, meaning that the true effect was probably markedly different from the estimated effect.

No straightforward comparison between our findings and those of earlier systematic reviews, in which meta-analysis was not employed [7,37], is possible. For example, the WHO review narratively summarized existing research and found low quality evidence for an association between road traffic noise and LBW, PTB, and SGA [7]. However, it included only two [18,22] of the seven studies considered here, and also included time-series studies. Indeed, there has been one previous attempt for a meta-analysis [8], but it focused on occupational noise exposure, which is not relevant to the present review.

One potential caveat in the WHO review was the rating of bias. The scoring criteria employed by Nieuwenhuijsen et al. [7] were limited and did not reflect domains such as response rate and sample selection. Our updated bias criteria included selection of participants as a potential source of bias [4]. Another deficiency was that the key confounding factors that one would expect a study to adjust for were not a priori defined and the quality of their assessment was not considered. Nieuwenhuijsen et al. [7] did not make it explicitly clear what they meant by the quality criterion “careful consideration of confounders” and which confounding factors were considered essential. That limited the transparency of their method. We view this feature as a more general caveat in the field. Currently, most primary studies on noise and birth outcomes adjust for largely the same confounder set, and it is implicitly assumed that “more is better”. Studies are deemed “less biased” owing to the richness of the covariates considered, however, that may lead to over-adjustment and actually bias the effect towards null. We reckon that this practice is mainly due to limited a priori rationale for ranking specific confounders according to their importance for the respective pregnancy outcome. Although we cannot provide a definitive solution here, we propose an alternative scoring protocol for the bias arising from the factors believed to confound the association in question. We propose that maternal age, education/socioeconomic position, smoking/alcohol use, and gestational age (when the outcome is birth weight or LBW) are the most important confounders [38,39,40]. However, although Smith et al. [10] adjusted for tobacco use and a deprivation index, those data were only available at area-level and may be subject to the ecological fallacy without complements at the individual-level [41]. Furthermore, potential effect modification may be masked by aggregation on factors like education/socioeconomic position in the study itself [42,43].

Exposure misclassification in included studies also merits consideration. In most studies, standard engineering models for noise and air pollution assessment were used, but description of traffic data used for calculation and its completeness was partially available. Furthermore, the methods used for propagation modelling cannot be easily compared and raise some questions. For example, the minimum noise level of 6.2 dB(A) in Gehring et al.’s study [18] seemed unreliable because prediction below 30 dB(A) is below the background level. Exposure misclassification could be one explanation of the major contribution of that study to heterogeneity in the birth weight model. However, after re-analyzing their data excluding exposure extremes, Gehring et al. did not observe any substantive change in the main findings [18]. When additional studies become available for meta-analysis, effect modification by quality of noise data should be investigated (i.e., quality of input data, availability of validation data, whether bedroom location, floor height, and window-opening habits were considered) [21]. The more detailed consideration of noise assessment quality as a potential source of bias in our meta-analysis (Table A1) did not lead us to a different conclusion. Hence, the present meta-analysis might not have had sufficient statistical power to tackle that issue.

Overall, we believe that these issues with the WHO evidence review [7] could have been avoided had the WHO commissioned the publication of systematic review protocols before the actual systematic reviews were conducted—something that was recently done for the WHO/ILO joint methodology for estimating the work-related burden of disease and injury from occupational noise exposure [44]. At this point, it is clearly beyond the scope of the present study to establish the superiority of one set of bias criteria over another; however, it is our belief that the time has come for development of standardized bias scoring checklists tailored specifically to studies on environmental noise and health outcomes. This becomes increasingly important with the introduction of new estimators in noise and health meta-analysis [21,44].

### 4.2. Strengths and Limitations

Our systematic review has several strengths. It included additional influential studies published after the WHO review was completed. Moreover, we were able to obtain useful data from Markevych et al.’s study [15], which was missed by the WHO review because relevant data were not reported. The number of estimates per outcome (≥5) in our review exceeded the number of studies included in the majority of meta-analyses on pregnancy outcomes listed in the Cochrane Database of Systematic Reviews [45]. We conducted multiple sensitivity analyses, such as identifying potentially influential studies and evaluating the confounding effect of air pollution.

Another strength is that we reported results under two estimators—the RE model, which readers and experts have grown to expect, and the QE model, which outperforms the RE model in the presence of high between-study heterogeneity. In fact, the QE model is already making its way in the field of environmental noise and health, and the recent WHO/ILO protocol for systematic reviews on occupational noise and cardiovascular disease made room for the possibility of supplementing the RE model with an alternative estimator under the QE model [44]. We see this as a precursor of a much needed paradigm shift. Furthermore, incorporating information on study quality in meta-analysis weights has been recommended over quality stratification, which can induce a spurious association between effect size and precision within stratum (collider-stratification bias) [46]. Given that bias scores are subjective to some degree and different scoring systems may yield different results, we compared our main findings against those based on an alternative set of bias criteria.

This work is not without limitations though. First, although we pooled a decent number of estimates per outcome, with less than 10 estimates we could not conduct meta-regressions and subgroup meta-analyses to determine whether specific study characteristics acted as effect modifiers. Second, we only considered 24-h noise exposure and did not find enough data to link birth outcomes to night-time noise. Third, only a handful of studies considered trimester-specific noise exposure, thus prohibiting time-window-specific meta-analyses. Fourth, the studies we pooled together differed in terms of outcome definition—some considered only term births, while others also included preterm births. Next, since most studies reported linear exposure-response associations, we could not test for a threshold effect, which would be important for recommending safe exposure limits. Finally, our literature searches were limited to papers published in English. Overall, most of these caveats are not limitations of our systematic review per se, but rather reflect methodological shortcomings in the respective literature.

## 5. Conclusions

We found “moderate” quality evidence that increasing maternal exposure to road traffic noise during pregnancy may be associated with lower birth weight of her newborn. The evidence on low birth weight, small for gestational age, and preterm birth was of “very low” quality and did not indicate any significant effect of noise. Sensitivity analyses including air pollution did not change the overall findings.

## Figures and Tables

**Table 1:** Descriptive characteristics of the studies included in the systematic review.

Study	Country	Design	Sample	Birth Outcomes	Noise Exposure	Adjustments in Main Model
Gehring et al. [18]	Canada	Cohort (1999/02)	68,238 singleton live births	Term BW, term LBW, SGA, PTB; Medical records	Modelled road traffic L_den_ at post code-level; Entire pregnancy; 60.2 ± 5.3 dB (6.2–89)	Sex, parity, nationality, maternal age, smoking, education, income, year and month, gestational week (for BW)
Dadvand et al. [19]	Spain	Cohort (2001/05)	6438 singleton term live births	Term LBW, SGA; Medical records	Modelled road traffic L_den_ within 50 m of the residence; Entire pregnancy; 68.7 ± 6.7 dB	Area-level SES, ethnicity, maternal education, marital status, age, smoking, alcohol, BMI, diabetes, infection, parity, infant sex, season and year
Markevych et al. [15]	Germany	Cross-sectional (1996/99)	1818 singleton term live births with normal BW	Term BW; Medical records	Modelled road traffic L_den_ at most exposed façade (noise barriers considered); Data available after delivery (2007); 53.8 ± 9 dB	Maternal age, education, smoking, infant sex, season and year, study area
Hjortebjerg et al. [22]	Denmark	Cohort (1996/02)	75,166 singleton term live births	Term BW; Medical records	Modelled road traffic L_den_ at most exposed façade; Entire pregnancy; 57.6 dB (49.4 – 69.7)	Gestational age, maternal age, BMI, height, parity, education, income, smoking, alcohol, city, other noise sources, NO_2_, infant sex, season
Barba-Vasseur et al. [9]	France	Case-control, 4:1 (2005/09)	1191 singleton births	PTB; Medical records	Modelled all-source L_den_ at each floor and façade; Data available at delivery; ≈ 55 dB (40–70)	Maternal age, parity, smoking
Smith et al. [10]	United Kingdom	Cohort (2006/10)	540,365/471,489 singleton term births	Term BW, Term SGA, Term LBW; Medical records	Modelled road traffic L_Aeq,16hr_ at most exposed façade; Data available at delivery; 58.1 ± 5.2 dB (54.7–86)	Infant sex, maternal age, ethnicity, birth registration type, area-level tobacco expenditure, Carstairs quintile, gestational age (for BW/LBW), season of birth, year, area
Wallas et al. [11]	Sweden	Cohort (1994/96)	4089 live births	LBW, PTB; Medical records	Modelled road traffic L_den_ at most exposed façade; Entire pregnancy; 52.1 dB (25–77.4)	Parental occupation, maternal smoking, BMI, municipality
Nieuwenhuijsen et al. [12]	United Kingdom, France, Spain, Lithuania, Norway, Greece	Cohort	31,458 singleton live term births	Term BW; Medical records	Modelled road traffic L_den_; Data available at delivery; 45% imputed values; 54.4 ± 7.2 dB	City, gestational age, infant sex, maternal age, education, parity, height and weight, smoking, season
Dzhambov et al. UIT [13]	Austria	Cross-sectional (1985/89)	573 live births	BW, LBW, SGA, PTB; Medical records	Modelled road traffic L_dn_ (calibration measurements); Data available after delivery (1997); 46.36 ± 13.8 dB	Infant sex, maternal age, marital status, education, smoking, gestational age (for LBW and BW), duration of residence before conception, house type
Dzhambov et al. BBT [13]	Austria, Italy	Cross-sectional (1992/98)	518 live births	BW, LBW, SGA, PTB; Medical records	Modelled road traffic L_den_ (calibration measurements); Data available after delivery (2003/04); 49.66 ± 14.3 dB	Infant sex, maternal age, education, smoking, gestational age (for LBW and BW), duration of residence before conception, house type

BBT: Brenner Base Tunnel, BW: Birth weight, BMI: Body mass index, L_Aeq,16hr_: Daytime equivalent noise level, LBW: Low birth weight, L_den_: Day-evening-night noise level, L_dn_: Day-night noise level, NO_2_: Nitrogen dioxide, PTB: Preterm birth, SES: Socioeconomic status, SGA: Small for gestational age, UIT: Unterinntal.

**Table 2:** Study quality scores based on the WHO evidence review bias criteria.

Study	Publication Type	Study Design	Noise Exposure	Birth Outcomes	Confounding Factors	Statistics	Bias	Overall Bias
Gehring et al. [18]	1	2	3	2	3	1	1	13 (low)
Dadvand et al. [19]	1	2	3	2	3	1	1	13 (low)
Markevych et al. [15]	1	0	3	2	1	1	0 ^1^	8 (unclear)
Hjortebjerg et al. [22]	1	2	3	2	3	1	1	13 (low)
Barba-Vasseur et al. [9]	1	2	3	2	1	1	0 ^2^	10 (low)
Smith et al. [10]	1	2	3	2	3	1	0 ^2,3^	12 (low)
Wallas et al. [11]	1	2	3	2	1	1	1	11 (low)
Nieuwenhuijsen et al. [12]	1	2	3	2	3	1	0 ^2,4^	12 (low)
Dzhambov et al. UIT [13]	1	0	3	2	1	1	0 ^1^	8 (unclear)
Dzhambov et al. BBT [13]	1	0	3	2	1	1	0 ^1^	8 (unclear)

Bias domains scoring: Publication type (0 = not peer reviewed, 1 = peer reviewed article), study design (1 = ecological, 2 = case control or cohort study, 3 = randomized control trial, 0 = other), noise exposure assessment (3 = objective assessment with noise measurements or noise calculations), assessment of the birth outcomes (1 = subjective assessment by report of mother, 2 = objective), confounding factors (0 = no confounding factors considered, 1 = confounding factors considered but some key confounders omitted, 3 = careful consideration of confounders), statistics (0 = flaws in or inappropriate statistical testing or interpretation of statistical tests that may have affected results, 1 = appropriate statistical testing and interpretation of tests), bias (0 = other study design or conduct issues that may have led to bias, 1 = no other serious study flaws). Interpretation of the total bias score: ≥10—low risk of bias, 6–9—unclear risk of bias, ≤5—high risk of bias. ^1^ Noise exposure data only available at a later point in time after pregnancy. ^2^ No information on maternal residential history during pregnancy. ^3^ Lack of individual-level data on important confounders. ^4^ A lot of imputed missing values in the noise exposure variable.

**Table 3:** Effect of a 10 dB(A) increase in road traffic noise on birth outcomes under the quality effects model in different adjustment scenarios.

Outcome (Model)	*N*	*β*/OR	95% CI	*I^2^* (%)
**Birth weight**				
Crude	5	−18.18	−44.53, 8.18	91
Main	6	−8.72	−25.20, 7.76	89
Air pollution-adjusted	6	−6.41	−24.11, 11.29	91
**Low birth weight**				
Crude	4	1.28	1.13, 1.46	48
Main	7	1.06	0.91, 1.23	49
Air pollution-adjusted	5	1.01	0.89, 1.14	37
**Small for gestational age**				
Crude	4	1.04	0.89, 1.22	91
Main	5	1.02	0.86, 1.21	90
Air pollution-adjusted	4	1.01	0.90, 1.15	84
**Preterm birth**				
Crude	4	1.02	0.96, 1.09	0
Main	5	1.00	0.79, 1.27	69
Air pollution-adjusted	3	1.00	0.79, 1.26	39

*β*: Unstandardized linear regression coefficient, *N*: Number of estimates in the model, NO_2_: Nitrogen dioxide, OR: Odds ratio, CI: Confidence interval, *I^2^*: Heterogeneity statistic. Studies used nitrogen dioxide as a proxy for traffic-related air pollution, except for Dadvand et al. [19] who adjusted for particulate matter.

## Appendix A

**Table A1:** Modified bias criteria and scoring for studies included in the systematic review.

Bias Criteria
**Publication type**
0 = Not peer reviewed
1 = Peer reviewed article
**Study design**
0 = Ecological
1 = Cross-sectional
2 = Case control
3 = Cohort study
**Selection of participants**
0 = No random sampling OR response rate less than 60% OR attrition rate higher than 20% OR no information provided
3 = Participants randomly sampled from a known population AND response rate higher than 60%/whole source population sampled AND attrition rate less than 20% in follow-up studies
**Noise exposure quality**
0 = Subjective method
1 = Objective method, low accuracy (e.g., land-use regression model, missing values)
2 = Objective method, limited accuracy or validity (e.g., postcode/street-level exposure, modelling only/no measurements, no data on floor, noise barriers, poor data on traffic)
3 = Objective method, accurate and valid (e.g., modelling and measurements, traffic evaluation)
**Noise exposure timeframe**
1 = Timeframe outside pregnancy and delivery
2 = Exposure at the date of delivery
3 = Exposure during pregnancy
**Assessment of birth outcomes**
0 = Subjective assessment by report of mother
2 = Objective (e.g., from medical records)
**Confounding factors**
0 = None or only one important confounding factor considered (maternal age or smoking/alcohol or education/socioeconomic position)
1 = Confounding factors considered and at least two of the following are considered: Maternal age; smoking/alcohol; education/socioeconomic position; gestational age (for birth weight and LBW—not relevant if only term births are considered)
2 = Consideration of all of the above confounders
3 = Consideration of all of the above and maternal BMI
4 = Consideration of all of the above and at least one of the following: Ethnicity; marital status/single mother; obstetric history; season/temperature; urbanicity
**Statistical analysis**
0 = Flaws in or inappropriate statistical testing or interpretation of statistical tests that may have affected results
2 = Appropriate statistical testing and interpretation of tests
**Additional bias**
0 = Several other study design or conduct issues that may have led to bias
1 = One other serious study flaw
3 = No other study serious flaws

**Table A2:** Alternative study quality scores based on the modified bias criteria.

Study	Publication Type	Study Design	Selection of Participants	Noise Exposure Quality	Noise Exposure Timeframe	Birth Outcomes	Confounding Factors	Statistics	Bias	Overall Bias
Gehring et al. [18]	1	3	3	2 (postcode-level)	3	2	2	2	3	21
Dadvand et al. [19]	1	3	3	2 (street-level)	3	2	4	2	3	23
Markevych et al. [15]	1	1	0 (1818 of 9086)	2 (no floor)	1	2	2	2	3	14
Hjortebjerg et al. [22]	1	3	3	2 (no floor/barriers)	3	2	4	2	3	23
Barba-Vasseur et al. [9]	1	2	3	3 (floor data)	2	2	1	2	0 ^1,2^	16
Smith et al. [10]	1	3	3	2 (no floor/barriers)	2	2	2	2	0 ^1,3^	17
Wallas et al. [11]	1	3	0 (baseline response not reported)	2 (no floor/barriers)	3	2	1	2	3	17
Nieuwenhuijsen et al. [12]	1	3	0 (baseline response not reported)	1 (45% missing values)	2	2	4	2	1 ^1^	16
Dzhambov et al. UIT [13]	1	1	0 (573 of 1280)	2 (less precise than the BBT survey)	1	2	2	2	3	14
Dzhambov et al. BBT [13]	1	1	0 (518 of 1251)	3 (advanced scientific methods, high resolution)	1	2	2	2	3	15

Bias domains scoring: See Table A1. ^1^ No information on maternal residential history during pregnancy. ^2^ Limited representativeness owing to exclusion of pregnancies with a wide range of pathology. ^3^ Lack of individual-level data on important confounders.

**Table A3:** Comparison of meta-analysis study weights under the quality effects model depending on the use of WHO evidence review vs. modified bias scores.

Study	Birth Weight (Study Weight %)		LBW (Study Weight %)		SGA (Study Weight %)		PTB (Study Weight %)	
	WHO Scores	Alternative Scores	WHO Scores	Alternative Scores	WHO Scores	Alternative Scores	WHO Scores	Alternative Scores
Gehring et al. [18]	10.6%	12.6%	11.8%	13.7%	10.4%	13.6%	79.9%	79.8%
Dadvand et al. [19]	-	-	2.9%	6.1%	2.4%	6.9%	-	-
Markevych et al. [15]	1.2%	3.0%	-	-	-	-	-	-
Hjortebjerg et al. [22]	11.4%	14.5%	-	-	-	-	-	-
Barba-Vasseur et al. [9]	-	-	-	-	-	-	5.5%	5.4%
Smith et al. [10]	66.5%	55.5%	78.7%	65.4%	85.0%	71.5%	-	-
Wallas et al. [11]	-	-	2.9%	5.0%	-	-	7.6%	7.2%
Nieuwenhuijsen et al. [12]	8.7%	8.7%	1.3%	3.2%	-	-	-	-
Dzhambov et al. [13] – UIT	0.8%	2.7%	1.1%	3.1%	1.1%	3.8%	3.4%	3.6%
Dzhambov et al. [13] – BBT	0.8%	2.9%	1.4%	3.6%	1.1%	4.1%	3.5%	4.0%

LBW: Low birth weight, PTB: Preterm birth, SGA: Small for gestational age.

## References

[1] Guski R., Schreckenberg D., Schuemer R. (2017). WHO Environmental Noise Guidelines for the European Region: A Systematic Review on Environmental Noise and Annoyance. Int. J. Environ. Res. Public Health.

[2] Clark C., Paunovic K. (2018). WHO Environmental Noise Guidelines for the European Region: A Systematic Review on Environmental Noise and Quality of Life, Wellbeing and Mental Health. Int. J. Environ. Res. Public Health.

[3] Basner M., McGuire S. (2018). WHO Environmental Noise Guidelines for the European Region: A Systematic Review on Environmental Noise and Effects on Sleep. Int. J. Environ. Res. Public Health.

[4] Van Kempen E., Casas M., Pershagen G., Foraster M. (2018). WHO Environmental Noise Guidelines for the European Region: A Systematic Review on Environmental Noise and Cardiovascular and Metabolic Effects: A Summary. Int. J. Environ. Res. Public Health.

[5] Jarosińska D., Héroux M.-È., Wilkhu P., Creswick J., Verbeek J., Wothge J., Paunović E. (2018). Development of the WHO Environmental Noise Guidelines for the European Region: An Introduction. Int. J. Environ. Res Public Health.

[6] WHO (2018). Environmental Noise Guidelines for the European Region.

[7] Nieuwenhuijsen M.J., Ristovska G., Dadvand P. (2017). WHO Environmental Noise Guidelines for the European Region: A Systematic Review on Environmental Noise and Adverse Birth Outcomes. Int. J. Environ. Res Public Health.

[8] Dzhambov A.M., Dimitrova D.D., Dimitrakova E.D. (2014). Noise exposure during pregnancy, birth outcomes and fetal development: Meta-analyses using quality effects model. Folia Med..

[9] Barba-Vasseur M., Bernard N., Pujol S., Sagot P., Riethmuller D., Thiriez G., Houot H., Defrance J., Mariet A.S., Luu V.P. (2017). Does low to moderate environmental exposure to noise and air pollution influence preterm delivery in medium-sized cities?. Int. J. Epidemiol..

[10] Smith R.B., Fecht D., Gulliver J., Beevers S.D., Dajnak D., Blangiardo M., Ghosh R.E., Hansell A.L., Kelly F.J., Anderson H.R. (2017). Impact of London’s road traffic air and noise pollution on birth weight: Retrospective population based cohort study. BMJ.

[11] Wallas A., Ekström S., Bergström A., Eriksson C., Gruzieva O., Sjöström M., Pyko A., Ögren M., Bottai M., Pershagen G. (2019). Traffic noise exposure in relation to adverse birth outcomes and body mass between birth and adolescence. Environ. Res..

[12] Nieuwenhuijsen M.J., Agier L., Basagaña X., Urquiza J., Tamayo-Uria I., Giorgis-Allemand L., Robinson O., Siroux V., Maitre L., de Castro M. (2019). Influence of the Urban Exposome on Birth Weight. Environ. Health Perspect..

[13] Dzhambov A.M., Markevych I., Lercher P. (2019). Associations of residential greenness, traffic noise, and air pollution with birth outcomes across Alpine areas. Sci. Total Environ..

[14] Moher D., Liberati A., Tetzlaff J., Altman D.G., the PRISMA Group (2009). Preferred reporting items for systematic reviews and meta-analyses: The PRISMA statement. Ann. Int. Med..

[15] Markevych I., Fuertes E., Tiesler C.M., Birk M., Bauer C.P., Koletzko S., von Berg A., Berdel D., Heinrich J. (2014). Surrounding greenness and birth weight: Results from the GINIplus and LISAplus birth cohorts in Munich. Health Place.

[16] Arroyo V., Díaz J., Ortiz C., Carmona R., Sáez M., Linares C. (2016). Short term effect of air pollution, noise and heat waves on preterm births in Madrid (Spain). Environ. Res..

[17] Arroyo V., Díaz J., Carmona R., Ortiz C., Linares C. (2016). Impact of air pollution and temperature on adverse birth outcomes: Madrid, 2001–2009. Environ. Pollut..

[18] Gehring U., Tamburic L., Sbihi H., Davies H.W., Brauer M. (2014). Impact of noise and air pollution on pregnancy outcomes. Epidemiology.

[19] Dadvand P., Ostro B., Figueras F., Foraster M., Basagaña X., Valentín A., Martinez D., Beelen R., Cirach M., Hoek G. (2014). Residential Proximity to Major Roads and Term Low Birth Weight: The Roles of Air Pollution, Heat, Noise, and Road-adjacent Trees. Epidemiology.

[20] Babisch W. (2008). Road traffic noise and cardiovascular risk. Noise Health.

[21] Dzhambov A.M., Dimitrova D.D. (2018). Residential road traffic noise as a risk factor for hypertension in adults: Systematic review and meta-analysis of analytic studies published in the period 2011–2017. Environ. Pollut..

[22] Hjortebjerg D., Andersen A.M.N., Ketzel M., Pedersen M., Raaschou-Nielsen O., Sørensen M. (2016). Associations between maternal exposure to air pollution and traffic noise and newborn’s size at birth: A cohort study. Environ. Int..

[23] Vienneau D., Eze I.C., Probst-Hensch N., Rȍȍsli M. Association between transportation noise and cardio-metabolic diseases: An update of the WHO meta-analysis. Proceedings of the 23rd International Congress on Acoustics.

[24] Brockwell S.E., Gordon I.R. (2007). A simple method for inference on an overall effect in meta-analysis. Stat. Med..

[25] Cornell J.E., Mulrow C.D., Localio R., Stack C.B., Meibohm A.R., Guallar E., Goodman S.N. (2014). Random-effects meta-analysis of inconsistent effects: A time for change. Ann. Intern. Med..

[26] Doi S.A., Barendregt J.J., Khan S., Thalib L., Williams G.M. (2015). Advances in the meta-analysis of heterogeneous clinical trials I: The inverse variance heterogeneity model. Contemp. Clin. Trials..

[27] Doi S.A., Barendregt J.J., Khan S., Thalib L., Williams G.M. (2015). Simulation Comparison of the Quality Effects and Random Effects Methods of Meta-analysis. Epidemiology.

[28] Doi S.A.R., Furuya-Kanamori L., Thalib L., Barendregt J.J. (2017). Meta-analysis in evidence-based healthcare: A paradigm shift away from random effects is overdue. Int. J. Evid. Based Healthc..

[29] Furuya-Kanamori L., Barendregt J.J., Doi S.A.R. (2018). A new improved graphical and quantitative method for detecting bias in meta-analysis. Int. J. Evid. Based Healthc..

[30] Barendregt J.J., Doi S.A. (2016). MetaXL User Guide: Version 5.3..

[31] Furuya-Kanamori L., Doi S.A. (2016). Angry Birds, Angry Children, and Angry Meta-Analysts: A Reanalysis. Perspect. Psychol. Sci..

[32] Guyatt G.H., Oxman A.D., Vist G.E., Kunz R., Falck-Ytter Y., Alonso-Coello P., Schünemann H.J. (2008). GRADE Working Group. GRADE: An emerging consensus on rating quality of evidence and strength of recommendations. BMJ.

[33] Hystad P., Davies H.W., Frank L., Van Loon J., Gehring U., Tamburic L., Brauer M. (2014). Residential greenness and birth outcomes: Evaluating the influence of spatially correlated built-environment factors. Environ. Health Perspect..

[34] Wu T.N., Chen L.J., Lai J.S., Ko G.N., Shen C.Y., Chang P.Y. (1996). Prospective study of noise exposure during pregnancy on birth weight. Am. J. Epidemiol..

[35] Cusack L., Sbihi H., Larkin A., Chow A., Brook J.R., Moraes T., Mandhane P.J., Becker A.B., Azad M.B., Subbarao P. (2018). CHILD Study Investigators. Residential green space and pathways to term birth weight in the Canadian Healthy Infant Longitudinal Development (CHILD) Study. Int. J. Health Geogr..

[36] Weyde K.V., Krog N.H., Oftedal B., Magnus P., White R., Stansfeld S., Øverland S., Aasvang G.M. (2018). A Longitudinal Study of Road Traffic Noise and Body Mass Index Trajectories from Birth to 8 Years. Epidemiology.

[37] Ristovska G., Laszlo H.E., Hansell A.L. (2014). Reproductive Outcomes Associated with Noise Exposure—A Systematic Review of the Literature. Int. J. Environ. Res. Public Health.

[38] Kramer M.S. (2003). The epidemiology of adverse pregnancy outcomes: An overview. J. Nutr..

[39] De Bernabé J.V., Soriano T., Albaladejo R., Juarranz M., Calle M.E., Marti’nez D., Domi´nguez-Rojas V. (2004). Risk factors for low birth weight: A review. Eur. J. Obstet. Gynecol. Reprod. Biol..

[40] Skrivankova V., Zwahlen M., Adams M., Low N., Kuehni C., Egger M. (2018). Spatial epidemiology of gestational age and birth weight in Switzerland: Census-based linkage study. BioRxiv.

[41] Wakefield J. (2008). Ecologic studies revisited. Annu. Rev. Public Health.

[42] Greenland S. (2001). Ecologic versus individual-level sources of bias in ecologic estimates of contextual health effects. Int. J. Epidemiol..

[43] Greenland S. (1992). Divergent biases in ecologic and individual-level studies. Stat. Med..

[44] Teixeira L.R., Azevedo T.M., Bortkiewicz A., da Silva D.T.C., de Abreu W., de Almeida M.S., de Araujo M.A.N., Gadzicka E., Ivanov I.D., Leppink N. (2019). WHO/ILO work-related burden of disease and injury: Protocol for systematic reviews of exposure to occupational noise and of the effect of exposure to occupational noise on cardiovascular disease. Environ. Int..

[45] Davey J., Turner R.M., Clarke M.J., Higgins J.P.T. (2011). Characteristics of meta-analyses and their component studies in the Cochrane Database of Systematic Reviews: A cross-sectional, descriptive analysis. BMC Med. Res. Methodol..

[46] Stone J., Gurunathan U., Glass K., Munn Z., Tugwell P., Doi S.A.R. (2019). Stratification by quality induced selection bias in a meta-analysis of clinical trials. J. Clin. Epidemiol..

